# Fidelity

**Published:** 1864-03

**Authors:** 


### FIDELITY.

SOME four years ago, a Baltimore gentleman had large interests at stake in St. Petersburgh, which required prompt and very close attention. Among all he knew, there was one man who seemed to him to possess the requisites for managing all matters faithfully, justly, and well; upon this individual he called, and explained to him at length the nature of the business, and the judgment and discretion requisite in bringing it to a satisfactory adjustment, concluding by saying: "If you are willing to go, I will give you twenty-five hundred dollars a month, but I wish you to take your time; do not hurry away a day sooner than is requisite to fully arrange all details and close the business in such a way, that any trouble hereafter shall be in a measure impossible." "I will go," said Mr. L. He was absent many months; and on his return, gave the fullest explanation to his employer, who, on gathering up his papers, expressed his satisfaction as to the manner in which his agent had acted, and handed him a check, which Mr. L. put in his pocket, without looking at it, supposing it was the amount due him according to the original understanding. But when he returned to his family he found, on reading the paper, that it was a token (irrespective of the original agreement) of Mr. W.'s appreciation of "fidelity" to an important trust, in the shape of a check for fifty thousand dollars. This morning's paper, of Thursday, August twenty-first, 1862, announces that a young man, the confidential clerk of a gentleman of wealth in New-York, had been intrusted by his employer with the duty of collecting several bank checks of several thousand dollars each. The young man did not return that day. But having acted with the strictest fidelity to the interests of the house for some years, no suspicions were harbored. When, however, he was not found at his place the next morning, some misgivings were slowly awakened; and, investigation discovered that the money had been collected, and that the unfaithful clerk had left the city. He was followed by the officers of the law, who found him secreted in the upper room of a hotel in a distant place in the interior of the State. He was returned to the city in irons, and confined in a felon's cell, and awaits the fearful punishment of wrong-doing. He said to the officers, that as soon as he left the city he became so nervous and conscience-stricken, that he did not know what course to pursue, and wandered around from place to place, without aim or end; the miserable victim of unappeasable remorse. In the former case, fidelity to trusts committed, has enriched a whole family for life; there being "thrown in" the sweet and comforting reflection, that their fortune was owing to a father's manly fidelity; the other, for the want of it, begins life at twenty-three, behind the bars of a jail, with no other rational prospect than that of suffering under the writhings of an outraged conscience, as long as life endures. The former will live in the serene contemplation of duty done, so promotive of health and happiness and a good old age; while the other, under the wasting influence of unavailing regrets, of sharp-pointed memories, and of bitter remorse, will doubtless sink into a premature grave, where body and memory will rot together.

